# Knowledge, Attitude, Awareness, and Barriers Toward Influenza Vaccination Among Medical Doctors at Tertiary Care Health Settings in Peshawar, Pakistan–A Cross-Sectional Study

**DOI:** 10.3389/fpubh.2018.00173

**Published:** 2018-06-27

**Authors:** Iftikhar Ali, Muhammad Ijaz, Inayat U. Rehman, Afaq Rahim, Humera Ata

**Affiliations:** ^1^Department of Pharmacy, University of Swabi, Swabi, Pakistan; ^2^Department of Pharmacy Services, Northwest General Hospital and Research Center, Peshawar, Pakistan; ^3^Paraplegic Center, Peshawar, Pakistan; ^4^Department of Medicine, Gajju Khan Medical College, Swabi, Pakistan; ^5^School of Pharmacy, Monash University Malaysia, Bandar Sunway, Malaysia; ^6^Department of Pharmacy, Abdul Wali Khan University Mardan, Mardan, Pakistan; ^7^Department of Medicine, Khyber Teaching Hospital, Peshawar, Pakistan; ^8^Department of International Maternal and Child Health, Uppsala University, Uppsala, Sweden

**Keywords:** Healthcare professionals, medical doctors, attitude, awareness, influenza, influenza vaccination, knowledge, Pakistan

## Abstract

**Objective:** This study intends to evaluate the knowledge, attitude and awareness of medical doctors toward influenza vaccination and the reasons for not getting vaccinated.

**Methods:** A cross-sectional study was carried out among medical doctors in three major tertiary care health settings in Peshawar, Khyber Pakhtunkhwa (KP), Pakistan. A web-based, pre-tested questionnaire was used for data collection.

**Results:** A total of (*n* = 300) medical doctors were invited, however only (*n* = 215) participated in the study with a response rate of 71.7%. Among the participants, 95.3% (*n* = 205) were males with a mean age of 28.67 ± 3.89 years. By designation, 121(56.3%) were trainee medical officers and 40 (18.6%) were house officers. The majority 102(47.4%) had a job experience of 1–2 years. Of the total sample, 38 (17.7%) doctors reported having received some kind of vaccination, whereas only 19 (8.84%) were vaccinated against influenza. The results identified that the major barriers toward influenza vaccinations included (1) Unfamiliarity with Influenza vaccination availability (Relative Importance Index RII = 0.830), (2) Unavailability of Influenza vaccines due to lack of proper storage area in the institution (RII = 0.634), (3) Cost of vaccine (RII = 0.608), and (4) insufficient staff to administer vaccine (RII = 0.589). Additionally, 156 (72.6%) of doctors were not aware of the influenza immunization recommendation and guidelines published by the Advisory Committee on Immunization Practices (ACIP) and Centre for Disease Control and Prevention (CDC). Physicians obtained a high score (8.27 ± 1.61) of knowledge and understanding regarding influenza and its vaccination followed by medical officers (8.06 ± 1.37). Linear Regression analysis revealed that gender was significantly associated with the knowledge score with males having a higher score (8.0± 1.39) than females (6.80 ± 1.61 β = −1.254 and CI [−2.152 to −0.355], *p* = 0.006).

**Conclusion:** A very low proportion of doctors were vaccinated against influenza, despite the published guidelines and recommendations. Strategies that address multiple aspects like increasing awareness and the importance of the influenza vaccine, the international recommendations and enhancing access and availability of the vaccine are needed to improve its coverage and health outcomes.

## Introduction

Influenza vaccination is the most efficient method of prevention of influenza virus infection and its complications (1, 2). Influenza is a common, highly contagious disease and healthcare professionals (HCPs) are at increased risk of getting infected and serve as carriers for transmission to other patients (3–5). Immunization against influenza virus not only reduces the risk of infection among HCPs, but also improves patient safety and reduces morbidity and mortality among vulnerable patients (3).

Influenza transmission within health care settings have been widely reported in medical literature (6). Therefore, both the World Health Organization (WHO) and the Strategic Advisory Group of Experts (SAGE) on Immunization recommend seasonal and pandemic influenza vaccination for HCPs (7, 8). Despite these guidelines and recommendations, vaccination coverage among HCPs has remained low, with little improvement during the pandemic of influenza A (H1N1) in 2009 (9). The scenario is more or less similar in European countries (10). Despite a variety of promotional campaigns and interventions, influenza vaccination acceptance among HCPs generally remains abysmal and most studies report poor adherence to this recommendation (11). The current rate of influenza vaccination for most health care settings remains at a meager 42% (12, 13). This low coverage might be attributable to apprehension regarding the effectiveness of the vaccine and concerns about adverse effects (12). Nevertheless, comprehending these barriers are important in controlling the low adherence to the recommendations for vaccination (7, 13, 14).

A literature search showed only one published study in 2016 among HCPs (Physicians, Pharmacists, Nurses, and Physiotherapists etc.) in private tertiary care settings in Peshawar on this subject which revealed poor knowledge of respondents toward influenza and its prevention (13). It also revealed misconceptions about influenza vaccine among the respondents (13). Therefore, this study fills the knowledge gap and aims to determine the vaccination rate and identify the factors influencing influenza vaccination among the doctors in tertiary care health settings in KP, Pakistan. The findings of this study can help in planning strategies to improve the situation and provide constructive steps toward future recommendations for vaccinating HCPs in Pakistan.

## Materials and methods

### Study design

A cross-sectional study among medical doctors was carried out in three major tertiary care hospitals in Peshawar, KP, Pakistan, from 20th March to 26th May 2016. The three tertiary care hospitals, namely: Hayatabad Medical Complex (HMC); Lady Reading Hospital (LRH); and Khyber Teaching Hospital (KTH), constitute the major public health facilities in Peshawar, the capital of Khyber Pakhtunkhwa. A previously validated (Cronbach's alpha = 0.87), self–administered, web-based questionnaire was circulated online using the Google forms platform. The questionnaire included 31 items (three sections) that collected information about the demographics (age, gender, designation, type of ward, job experience, and hospital), knowledge of influenza virus and vaccination, current practice and awareness of published guidelines. The link to the online survey was sent through email and popular social networks (Facebook, WhatsApp) to medical doctors. Those who followed the link were asked to affirm their agreement to participate in the survey. The study aimed to recruit at least 300 medical doctors in three tertiary care health settings, however 215 participated in this study. Due to the pilot nature of this study no formal sample size calculation was conducted.

### Ethics approval

The study protocol was approved by Bacha Khan Medical Complex (Teaching Hospital of Gajju Khan Medical College), Shah Mansoor, Swabi, KP, Pakistan (Reference Number, EC-5/6/17).

### Questionnaire items

A 31-item questionnaire was used to attain the objectives of the study. The questionnaire was adopted from existing literature addressing the same research question in other regions (12). The questionnaire comprised of three sections;

- The first section comprised of eight items aiming to inquire about the demographic information of doctors and their disclosure by asking whether they have been vaccinated against influenza or any other disease.
- The second section consisted of eight items, inquiring about the reasons for not receiving vaccination against influenza. A five-item Likert-scale was provided to choose the relevant response.
- The third section comprised of four items aiming to explore the doctors' general understanding about influenza vaccination.
- The last part, or section three, of the questionnaire was related to the knowledge of influenza and influenza vaccines. It comprised of 11 statements, a nominal scale of correct and incorrect was provided for the doctors' convenience to disclose their response (Data Sheet 1).

### Statistical analysis

For data analysis, SPSS®V 20.0 (IBM Corp., Armonk, NY, USA) was used. Frequency and Percentages were used for categorical variables. A Fisher exact test was applied to identify the significant factors hindering influenza vaccination. Additionally, to identify the top four barriers toward influenza vaccination among doctors, a Relative Importance Index (RII) (15, 16) was calculated for each of the eight statements. “RII is a commonly used method to obtain priority rankings of attributes and it is particularly useful where a structured questionnaire is used to solicit measurements that are subjective in nature” (17) The RIIs is calculated by the formula shown in Equation (1.0) (18).

(1.0) $$\begin{array}{r} \text{RII }=\;\left(1{\text{n}}_{1}+2{\text{n}}_{2}+3{\text{n}}_{3}+4{\text{n}}_{4}+5{\text{n}}_{5}\right)/5\text{N}\left(0\leq \text{RII}\leq 1\right) \end{array}$$

Where: *N* = Total number of respondent, 5 = highest weighted score (1, 2, 3, 4, 5) on Likert scale where: n1 = number of participants who selected “strongly disagree,” n2 = number of participants who selected “disagree,” n3 = number of participants who selected “do not know,” n4 = number of participants who selected “agree,” and n5 = number of participants who selected “strongly agree.” The score for each factor is calculated by summing up the scores given to it by the participants.

The mean item score was used to rank top four barriers. The 5-point Likert scale was converted to index for each barrier, which made it possible to cross-compare the relative importance of each of the barriers as supposed by the participants. The value of the RII ranges from 0 to 1 (17), a value closest to 1 ranked as the main barrier to influenza vaccination compared to others. For the 11 items regarding the knowledge (**Table 4**), scoring of the responses was done using 1 and 0 for correct and incorrect responses respectively. To identify the contributing factors affecting the knowledge score, linear regression analysis was conducted using score as the dependent variable while using age, gender, designation, and job experience as the independent variables. Moreover, Mann–Whitney and Kruskul Wallis test were used to closely notice the differences in knowledge among the groups. A *P*-value of < 0.05 was set significant for all of the statistical tests.

## Results

A total of *n* = 300 medical doctors were invited, however *n* = 215 participated in this study and the response rate was 71.7%. The mean age was 28.67 ± 3.89 years and the majority were males, *n* = 205 (95.3 %). By designation, 121(56.3%) were trainee medical officers, 40 (18.6%) were house officers and 36 (16.7%) medical officers. A majority of the doctors 102(47.4%) had a job experience of 1–2 years followed by 52 (24.2%) with 3–5 years job experience. Only 17.7% of the doctors had history of vaccination in the past 6–12 months for other diseases, of which influenza vaccine comprised 50% (Table 1).

**Table 1:** Demographics of Respondents (*N* = 215).

**Demographics**	**N (%)**
**Age**	
Range	24 years−65 years
Mean age ±SD	28.67 ± 3.89 years
**Gender**	
Male	205 (95.3%)
Female	10 (04.7%)
**Job experience**	
Less than 1 year	30 (14.0%)
1–2 years	102 (47.4%)
3–5 years	52 (24.2%)
6–10 years	31 (14.4%)
**Hospitals**	
Lady Reading Hospital, Peshawar	44 (20.5%)
Hayatabad Medical Complex, Peshawar	48 (22.3%)
Khyber teaching Hospital, Peshawar	123 (57.2%)
**Type of ward**	
Medicine	140 (65.1%)
Surgery	75 (34.9%)
**Designations**	
Trainee medical officer	121 (56.3%)
Medical Officer	36 (16.7%)
House Officer	40 (18.6%)
Registrar	07 (3.3%)
Physicians	11 (5.11%)
**Vaccination done in last 6–12 months against any disease**	
Yes	38 (17.7%)
No	114 (53.02%)
Never vaccinated in last 2-3 years' time	63 (29.30%)
**Vaccination done in last 6–12 months against influenza**	
Yes	19 (8.84%)
No	196 (91.16%)
**Name the disease for which you have done vaccination**	
Influenza	19 (50%)
Hepatitis	17 (44.74%)
Tetanus toxoid	01 (2.63%)
Anti-Rabies	01 (2.63%)

While evaluating the doctors' justifications for not being vaccinated against influenza, ~78.1% of the respondents disagreed that *influenza is not serious condition therefore not worth vaccinating*, however, 13.49%, believed that *it is not compulsory for HCPs to get vaccinated for influenza*. None of the statements was associated with uptake of vaccination against influenza. Upon calculation of the RII, the top four barriers identified were*: “Not everyone is familiar with Influenza vaccination availability at their institutions” (RII* = *0.830), “There is lack of proper storage area for vaccines that's why Influenza vaccines are not available in the institution” (RII* = *0.634)*, “*Influenza vaccine is costly that's why not purchased normally” (RII* = *0.608) and “There is insufficient staff to administer vaccine” (RII* = *0.589)* as shown in (Table 2).

**Table 2:** Reason for not vaccination against influenza.

**Statements**	**SDA**	**DA**	**DK**	**A**	**SA**	***P*-value**	**RII**	**Rank**
There is lack of proper storage area for vaccines that's why Influenza vaccines are not available in the institution	07 (3.3%)	40 (18.6%)	100 (46.5%)	46 (21.4%)	22 (10.2%)	0.454	0.634	2
It is not compulsory for health care professionals to get vaccinated for Influenza	58 (27.0%)	89 (41.4%)	39 (18.1%)	21 (9.8%)	8 (3.7%)	0.600	0.444	7
Influenza is not serious condition therefore not worth vaccinating against	50 (23.3%)	118 (54.9%)	18 (8.4%)	28 (13.0%)	1 (0.5%)	0.446	0.425	8
Influenza vaccine is costly that's why not purchased normally	14 (6.5%)	38 (17.7%)	100 (46.5%)	51 (23.7%)	12 (5.6%)	0.170	0.608	3
Not everyone is familiar with Influenza vaccination availability at their institutions	02 (0.9%)	09 (4.2%)	11 (5.1%)	126 (58.6%)	67 (31.2%)	0.725	0.830	1
There is insufficient staff to administer vaccine	12 (5.6%)	76 (35.3%)	49 (22.8%)	68 (31.6%)	10 (4.7%)	0.303	0.589	4
Side effects and safety concerns are hindering health care professionals to get vaccinated for influenza	14 (6.5%)	105 (48.8%)	55 (25.6%)	36 (16.7%)	5 (2.3%)	0.080	0.519	5
Due to needle fear I don't like to get vaccinated	29 (13.5%)	125 (58.1%)	11 (5.1%)	39 (18.1%)	11 (5.1%)	0.064	0.487	6

*Fischer exact test was applied; SDA, strongly disagree; DA, Disagree; DK, Don't Know; A, Agree; SA, strongly agree; RII, Relative importance index*.

Regarding the general understanding of doctors about influenza vaccine, 145(67.4%) responded that “*the influenza vaccine is effective in preventing the flu”*, while 116(54.0%) responded that “*CDC recommends that healthcare professionals receive the flu shot”*. About 165 (72.6%) participants were not “*aware of the guidelines published by the ACIP or CDC for influenza immunization”* and regarding the influenza vaccination schedule. A majority of 61.9% of the doctors thought that “*influenza vaccine is to be administered once in life”* (Table 3).

**Table 3:** Medical doctors' general understanding about the influenza vaccine.

**Statement**	***N* (%)**
**Do you think the influenza vaccine is effective in preventing the ‘flu?**	
Yes	145 (67.4%)
No	14 (6.5%)
Not Sure	56 (26.0%)
**Do you believe that the Centre for Disease Control (CDC) recommends that healthcare professionals receive the flu shot?**	
Yes	116 (54.0%)
No	23 (10.7%)
Not sure	76 (35.3%)
**Are you aware of the guidelines published by the Advisory Committee on Immunization Practices (ACIP) or CDC for influenza immunization?**	
Yes	34 (15.8%)
No	156 (72.6%)
Not sure	25 (11.6%)
**How often do you think the flu vaccine should be administered?**	
Every year	21 (9.8%)
Once in life	133 (61.9%)
Every 6 months	51 (23.7%)
Every 5 years	8 (3.7%)
Never	2 (0.9%)

Table 4 depicts the doctors knowledge toward influenza and influenza vaccine. It is revealed that 79(36.7%) of the doctors had a misconception regarding the composition of influenza vaccines; 129(60.0%) believed that the “*flu shot contains a live virus that may cause influenza*”. Moreover, a majority, 161(74.9%), believed that “*symptoms usually appear 8–10 days after a person is exposed to influenza*”. Nearly 38.1% believed that people “*with influenza can transmit the infection only after their symptoms appear”* and about 57(26.5%) believed “*HCPs can not spread influenza even when they are feeling well*”. About 62(28.8%) believed that “*nausea and vomiting, or diarrhea, are common symptoms observed during an influenza infection*”. In addition, 40(18.6%) of the doctors believe that Influenza is transmitted primarily by contact with blood and body fluids.

**Table 4:** Knowledge of medical doctors about influenza and the influenza vaccine.

**Statement**	**Correct**	**Incorrect**
Q1. Health care professionals are less susceptible to influenza infections than other people	17 (7.9%)	198 (92.1%)^**^
Q2. Influenza is transmitted primarily by coughing and sneezing	210 (97.7%)^**^	5 (2.3%)
Q3. Influenza is more serious than a “common cold	203 (94.4%) ^**^	12 (5.6%)
Q4. The signs and symptoms of influenza include fever, headache, sore throat, cough, nasal congestion, and aches and pains	213 (99.1%)^**^	2 (0.9%)
Q5. HCPs can spread influenza even when they are feeling well.	158 (73.48%)^**^	57 (26.5%)
Q6. People with influenza can transmit the infection only after their symptoms appear	82 (38.1%)	133 (61.9%)^**^
Q7. Influenza is transmitted primarily by contact with blood and body fluids	40 (18.6%)	175 (81.4%)^**^
Q8. The flu shot contains live viruses that may cause some people to get influenza	129 (60.0%)	86 (40.0%)^**^
Q9. Influenza vaccination does not work in some persons, even if the vaccine has the right mix of viruses	136 (63.3%)^**^	79 (36.7%)
Q10. Adults with influenza commonly experience nausea and vomiting or diarrhea	62 (28.8%)	153 (71.2%)^**^
Q11. Symptoms typically appear 8–10 days after a person is exposed to influenza	161 (74.9%)	54 (25.1%)^**^

* *Correct statement, Score range 1–11*.

Among the participants the knowledge and understanding about influenza vaccination did not differ significantly with physicians gaining a slightly higher score (8.27 ± 1.61) then medical officers (8.06 ± 1.37) and trainee medical officers (8.02 ± 1.39). Doctors with job experience of 6-10 years had better knowledge about influenza vaccination (8.19 ±1.30) compared to doctors with less than 1 year job experience (7.87 ±1.38). Additionally, male doctors possessed better knowledge (8.05 ±1.39, *P* = 0.003) than female doctors as shown (Table 5).

**Table 5:** Medical doctors score for knowledge about Influenza and the influenza vaccine against gender, designation, and job experience.

**Variable**	**Mean ± SD**	***p*-value**
**Gender**		0.003^*^^a^
Male	8.05 ± 1.39	
Female	6.80 ± 1.61	
**Designation**		0.469^b^
House Officer	7.95 ± 1.60	
Medical Officer	8.06 ± 1.37	
Trainee medical officer	8.02 ± 1.39	
Registrar	7.14 ± 1.39	
Physicians	8.27 ± 1.61	
**Job experience**		0.774^b^
Less than 1 year	7.87 ± 1.38	
1–2 years	7.89 ± 1.48	
3–5 years	8.15 ± 1.42	
6–10 years	8.19 ± 1.30	

a *Mann–Whitney test*.

b *Kruskul Wallis test*.

* *P-values < 0.05 was considered significant*.

Linear regression was applied using sum of 11 items score as the dependent variable and age, gender, designation and job experience as the independent variable. Overall, gender was found to be a significant predictor of the total knowledge score and males had a higher score than the females with β = −1.254 and CI [−2.152 to −0.355], *p* = 0.006; while age, job experience and designation were observed to be insignificant factors of the overall doctors' knowledge as shown in (Table 6).

**Table 6:** Factors affecting the Knowledge score of Medical doctors.

**Variable**	**Regression coefficient (β) [95% CI]**	**t**	**Std. error**	***p*-value**
Age	0.039[−0.009 to 0.088]	1.614	0.024	0.108
Gender	−1.254[−2.152 to −0.355]	−2.750	0.456	0.006
Designation	0.141[−0.0073 to 0.355]	1.299	0.108	0.195
Job experience	0.008[−0.186 to 0.202]	0.077	0.098	0.939

*Linear regression was applied using score as a dependent variable and age, gender, designation, and job experience as independent variable. P-values < 0.05 were considered statistically significant*.

## Discussion

Compliance of HCPs to influenza vaccination is a matter of intense debate. The present study is perhaps the first-of-its-kind to evaluate Pakistani medical doctors' knowledge, and attitude toward influenza vaccination in public sector tertiary care health settings in Peshawar, Khyber Pakhtunkhwa as a previous study in Pakistan investigated knowledge and attitude to influenza vaccination in all health care professionals in a private setup (13). It is evident from several published studies that vaccinating HCPs against influenza is an effective intervention to protect against infections, reduce its transmission to the patients and to decrease mortality and morbidity among the vulnerable patients (2, 13, 19). Vaccination also reduces absenteeism and improves the health status of HCPs (6, 11, 20, 21). However, this study reveals low rates of immunization against influenza among the doctors in tertiary care health settings of Peshawar, Pakistan, despite the ACIP and CDC recommendations and HCPs being at higher risk of infection. These findings are in line with some published studies that described a lower rate of influenza immunization among HCPs (13, 22). Moreover, influenza vaccination rate among doctors figured by the present study is, probably, the lowest among HCPs compared with available literature from different region of the world i.e., “Kuwait (67.2%) Oman (46.4%), Kingdom of Saudi Arabia [KSA (38.0%) and United Arab Emirates (UAE(24.7%)]” (12, 13).

The proportions of HCPs who have been vaccinated against influenza are quite low in Pakistan, which has been justified in a recent study among HCPs done in Peshawar to investigate knowledge, awareness and attitude toward influenza vaccination (13, 23). The low uptake of influenza immunization among practicing doctors in the present study seems to be related to unfamiliarity with influenza vaccination, cost and understanding. Our survey was unable to show a consistent positive correlation between HCPs' perception of efficacy of the influenza vaccine and the choice to be vaccinated. Conversely, our study showed that 67.4% of the study sample was aware that the vaccine was effective in preventing influenza with a low self-declared vaccination rate.

It is essential to understand the factors that affect HCPs compliance toward influenza vaccines. One study revealed that HCPs' fear of adverse reaction may act as one of the main barriers to vaccination (24). Moreover, it is also noticed that about 10–45% of HCPs feared getting vaccinated induced influenza (13, 25). Surprisingly, 06–58% of HCPs from technologically advanced countries like Switzerland and Canada believed that they were healthy and they preferred to have good natural defense toward influenza infection (25–28). Comparing the result of this study to that of developed countries, the fear of side effects was only disclosed by 19.1% of the respondents and the rest disagreed with the statement that side effect and safety concerns might hinder HCPs willingness to get a flu shot. Hofmann et al. showed that in some cases, approximately 54.0% of HCPs believed that they may suffer adverse drug reactions, which was the main reason for their reluctance to get vaccinated (24). In the current sample, it was revealed that the main barrier to influenza vaccination among HCPs is the unfamiliarity with Influenza vaccination availability, cost and lack of proper storage area for vaccine at their institutions. These findings are in agreement to the results reported in studies conducted by Khan TM et al. in Pakistan (13) and James, P B et al. in Sierra Leone (29) in which lack of awareness about the vaccine availability at their institution, high cost of the vaccine and lack of awareness were put forward by participants as barriers to influenza vaccinations. Other barrier to influenza vaccinations identified in this study included the fear of needles and the side-effects, lack of sufficient staff to administer vaccine and safety concerns associated with influenza vaccinations. Fear of needle and concerns about side-effects of the vaccine are also some of the reasons that have been reported in other studies (12, 13, 20). In the current study, about 23.2% of the respondents disclosed their unwillingness to vaccinations due to fear of needles. Similarly, a study by Hofmann et al. (24) reported 4–26% of HCPs with fear of injections. These knowledge gaps are essential to be addressed among Pakistani HCPs, and such wrong beliefs are perhaps due to the lack of any health campaigns among the HCPs to create awareness about influenza vaccination. Most HCPs had basic knowledge of the influenza vaccine and infection and most participating doctors were aware that people with asymptomatic influenza can transmit influenza (61.9%); a study conducted in Iran by Khazaeipour et al.'s reported similar beliefs among HCPs, with only 32.4% of HCPs stating that people with asymptomatic influenza can transmit influenza (30).

Furthermore, our data showed variable levels of knowledge about influenza vaccination. Nearly 97.7% of the doctors have a belief that Influenza is transmitted primarily by coughing and sneezing; broadly in line with other studies (13, 22, 31–33). A similar level of response (90.61%) was seen by Alshammari TM (12). Moreover, 99.1% have a proper understanding about the symptoms of influenza and 153(71.2%) expressed that symptoms such as nausea and vomiting or diarrhea are common in influenza infection among adults. Furthermore, 60.0% of all participants in this study shared a view that influenza vaccine is infectious and can cause influenza. Previous studies have presented various rates of belief amongst HCPs that the influenza vaccine can cause influenza infection (38.1–78%) (32–35). Only 15.8% of the doctors were familiar with the influenza immunization program for HCPs. A number of studies have described that in KSA, nearly 86% of the respondents were familiar with the recommendation of ACIP and in the 3 Middle-East countries: Kuwait, Oman, and UAE, nearly 49% of HCPs were knowledgeable of the CDC recommendations regarding seasonal influenza vaccination (1, 12, 36). We noted much lower perception of these guidelines in our study sample; around 72.6% of participating doctors were not aware of the CDC or ACIP recommendations. In our study, physicians were found to have a good knowledge of the influenza symptoms and vaccinations, followed by the medical officers. In addition, participants with a job experience of 6–10 years were seen to have a significantly better knowledge as compared to others.

Some other studies conducted in the Asian countries have also provided medium to low correlation of gender and age with the individual questions presented to evaluate attitudes and knowledge of HCPs toward influenza vaccination (13, 32, 36), though, when sum of knowledge items was tested to see the factors affecting the knowledge score, gender was found to be another significant factor affecting the knowledge score of doctors.

After assessing the attitude and knowledge toward vaccination, a foresighted level was observed among Pakistani doctors. Already the education level of the doctors is fair enough but still a robust plan is needed to be implemented at government level to highlight the need and importance of influenza vaccination and the government should work to include influenza vaccination in national immunization programs. Additionally, a regular monthly newsletter should be circulated by the ministry of health to all practicing HCPs in order to educate them and make them aware of the influenza vaccination availability at hospitals and free the cost for the general masses in order to cope with influenza spread, thus supporting good health retention in HCPs and stopping the spread of such diseases to patients.

## Conclusion

Despite the published guidelines and recommendations, a very low proportion of doctors in the three tertiary care health settings of Peshawar were vaccinated against influenza. The barriers and misconceptions about influenza and vaccines should be reduced through multiple strategies, which include organizing seminars on awareness regarding vaccinations. This is required to improve the understanding and general results. Pakistani health authorities need to inspire all HCPs to receive vaccination and improve their degree of compliance with recommendations for immunization against influenza by announcing it as mandatory for all HCP's working in health settings. Furthermore, implementation of an occupational health department in each tertiary hospital overseeing vaccination status would also help keep track of and ensure adequate compliance amongst HCPs.

## Author contributions

Conception and design: IA, MI. Data collection: MI, IA. Data analysis and interpretation, Results: IA, IR, HA. Manuscript drafting and writing: IA, MI, IR, AR. Language editing, appropriateness, critical revision: AR, HA, IA. All authors read and approved the final version of the paper.

### Conflict of interest statement

The authors declare that the research was conducted in the absence of any commercial or financial relationships that could be construed as a potential conflict of interest.

## Abbreviations

HCPs: Healthcare professionals
WHO: World Health Organization
SAGE: Strategic Advisory Group of Experts
HMC: Hayatabad Medical Complex
LRH: Lady Reading Hospital
KTH: Khyber Teaching Hospital
CDC: Centre for Disease Control and Prevention
ACIP: Advisory Committee on Immunization Practices.
