# Supplementary material for: Knowledge, Attitude, Awareness, and Barriers Toward Influenza Vaccination Among Medical Doctors at Tertiary Care Health Settings in Peshawar, Pakistan–A Cross-Sectional Study
Source: Front Public Health. 2018 Jun 27;6:173. doi: 10.3389/fpubh.2018.00173 (PMC6030373; doi:10.3389/fpubh.2018.00173)
Supplement: Supplementary file 1 [file Data_Sheet_1.docx]

**Appendix I**

**Knowledge, Attitude, Awareness and Barriers towards Influenza towards Influenza Vaccination among Medical Doctors at Tertiary Care Health Settings of Peshawar, Pakistan–A Cross-Sectional Study**

**1. Age (years)**

**__________**

**2. Gender**

- Male
- Female

**3. Job experience (years)**

- Less than 1 year
- 1-2 years
- 3-5 years
- 6-10 years

**4. Hospitals**

- Lady Reading Hospital, Peshawar
- Hayatabad Medical Complex, Peshawar
- Khyber teaching Hospital, Peshawar

**5. Type of ward**

- Medicine
- Surgery

**6. Designation**

- - Trainee medical officer
  - Medical Officer
  - House Officer
  - Registrar
  - Physicians

**7. in last 6-12 months, have you vaccinated yourself against any disease?**

- Yes
- No
- Never vaccinated in last 2-3 years’ time

**If yes to Q 7 please provide the name of vaccine**

_I._**_______________** _II._ **_________________**_III._ **_______________**

**8. In last 6-12 months have you vaccinated yourself against influenza?**

- Yes
- No
- Never vaccinated in last 2-3 years’ time

**9. There is lack of proper storage area for vaccines that’s why Influenza vaccines is not available in the institution**

Reasons for not vaccinating against influenza Kindly share your opinion

- Strongly Agree
- Agree
- Don't know
- Disagree
- Strongly Disagree

**10. It is not compulsory for health care professionals (HCPs) to get vaccinated for Influenza**

Reasons for not vaccinating against influenza Kindly share your opinion.

- Strongly Agree
- Agree
- Don't know
- Disagree
- Strongly Disagree

**11. Influenza is not serious condition therefore not worth vaccinating**

Reasons for not vaccinating against influenza Kindly share your opinion.

- - Strongly Agree
  - Agree
  - Don't know
  - Disagree
  - Strongly Disagree

**12. Influenza vaccines is costly that’s why not purchased normally**

Reasons for not vaccinating against influenza Kindly share your opinion**.**

- - Strongly Agree
  - Agree
  - Don't know
  - Disagree
  - Strongly Disagree

**13. Not everyone is familiar with Influenza vaccination**

Reasons for not vaccinating against influenza Kindly share your opinion**.**

- Strongly Agree
- Agree
- Don't know
- Disagree
- Strongly Disagree

**14. There is insufficient staff to administer vaccine**

Reasons for not vaccinating against influenza Kindly share your opinion**.**

- Strongly Agree
- Agree
- Don't know
- Disagree
- Strongly Disagree

**15. Side effects and safety concerns are hindering HCPs to get vaccinated for influenza**

Reasons for not vaccinating against influenza Kindly share your opinion**.**

- Strongly Agree
- Agree
- Don't know
- Disagree
- Strongly Disagree

**16. I don’t like needles**

Reasons for not vaccinating against influenza Kindly share your opinion**.**

- Strongly Agree
- Agree
- Don't know
- Disagree
- Strongly Disagree

**17. Do you think the influenza vaccine is effective in preventing the ‘flu?**

HCPs general understanding about the influenza vaccine. Kindly share your opinion**.**

- Yes
- No
- Not sure

**18. Do you believe that the Centre for Disease Control recommends that HCPs receive the flu shot?**

HCPs general understanding about the influenza vaccine. Kindly share your opinion**.**

- Yes
- No
- Not sure

**19. Are you aware of the guidelines published by the Advisory Committee on Immunization Practices (ACIP) or Centre for Disease Control for influenza immunization?**

HCPs general understanding about the influenza vaccine. Kindly share your opinion**.**

- Yes
- No
- Not sure

**20. How often do you think the flu vaccine should be administered?**

HCPs general understanding about the influenza vaccine

- Every 6 months
- Every year
- Every 5 years
- Once in a lifetime
- Never

**21. HCPs are less susceptible to influenza infections than other people**

Awareness of HCPs about Influenza and the Influenza Vaccine. Kindly share your opinion

- Correct
- Incorrect

**22. Influenza is transmitted primarily by coughing and sneezing**

Awareness of HCPs about Influenza and the Influenza Vaccine. Kindly share your opinion

- Correct
- Incorrect

**23. Influenza is more serious than a “common cold**

Awareness of HCPs about Influenza and the Influenza Vaccine. Kindly share your opinion

- Correct
- Incorrect

**24. The signs and symptoms of influenza include fever, headache, sore throat, cough, nasal congestion, and aches and pains,**

Awareness of HCPs about Influenza and the Influenza Vaccine. Kindly share your opinion

- Correct
- Incorrect

**25. HCPs can spread influenza even when they are feeling well**

Awareness of HCPs about Influenza and the Influenza Vaccine. Kindly share your opinion

- Correct
- Incorrect

**26. People with influenza can transmit the infection only after their symptoms appear**

Awareness of HCPs about Influenza and the Influenza Vaccine. Kindly share your opinion

- Correct
- Incorrect

**27. Influenza is transmitted primarily by contact with blood and body fluids**

Awareness of HCPs about influenza and the influenza vaccine. Kindly share your opinion

- Correct
- Incorrect

**28. The flu shot contains live viruses that may cause some people to get influenza**

Awareness of HCPs about Influenza and the Influenza Vaccine. Kindly share your opinion

- Correct
- Incorrect

**29. Influenza vaccination does not work in some persons, even if the vaccine has the right mix of viruses**

Awareness of HCPs about Influenza and the Influenza Vaccine. Kindly share your opinion

- Correct
- Incorrect

**30. Adults with influenza commonly experience nausea and vomiting or diarrhea**

Awareness of HCPs about Influenza and the Influenza Vaccine. Kindly share your opinion

- Correct
- Incorrect

**31. Symptoms typically appear 8–10 days after a person is exposed to influenza**

Awareness of HCPs about Influenza and the Influenza Vaccine. Kindly share your opinion

- Correct
- Incorrect
